# P-1121. Resistance-Driven Outbreak Detection and Etiologic Investigation for Hospital-Onset Pseudomonas aeruginosa Using Machine Learning on Electronic Health Records

**DOI:** 10.1093/ofid/ofaf695.1316

**Published:** 2026-01-11

**Authors:** Scott A Cohen, Massimiliano S Tagliamonte, Nicole M Iovine, Kathryn DeSear, KwangCheol Casey Jeong, Yuting Zhai, Marco Salemi, Mattia Prosperi, J Glenn Morris

**Affiliations:** University of Florida, Gainesville, FL; University of Florida, Gainesville, FL; University of Florida, Gainesville, FL; UF health Shands Hospital, Gainesville, Florida; University of Florida, Gainesville, FL; University of Florida, Gainesville, FL; University of Florida, Gainesville, FL; University of Florida, Gainesville, FL; University of Florida, Gainesville, FL

## Abstract

**Background:**

Hospital outbreak identification often lacks standardization and uses time-intensive surveillance, potentially delaying intervention against hospital-acquired pathogens. We combined space-time permutation analysis with machine learning to statistically identify hospital-onset *Pseudomonas aeruginosa* outbreaks and contributing etiologies.

**Methods:**

We retrospectively analyzed hospital-onset *P. aeruginosa* isolates (2016-2024) using electronic health records (EHR) at a single tertiary care center. Hospital-onset infections were defined as cultures collected more than two days after admission. Susceptibility profiles were standardized by imputing intrinsic resistance and classifying extensive (XDR) or multidrug-resistance (MDR). Space-time permutation analysis (modified WHONET-SatScan) identified resistance-based clusters, validated by whole-genome sequencing (WGS). For each cluster, we conducted case-control studies comparing identified cases with non-case *P. aeruginosa* during the same period. Feature selection for temporally scaled risk factors was performed using elastic net regularization.

**Results:**

Among 19,055 hospitalizations (11,112 patients) with *P. aeruginosa*, 6,557 (34.4%) were hospital-onset. Hospital-onset isolates were 1.9 (95% CI: 1.8–2.1) times more likely to be MDR and 2.2 (95% CI: 1.9–2.6) times more likely to be XDR compared to community-onset. Of 615 unique resistance profiles identified, 53% were susceptible to all antipseudomonal classes. Resistance to antipseudomonal cephalosporins was most common (30.2%). Space-time permutation analysis detected 12 unique clusters, none matching WGS-identified clusters. One cluster defined by cephalosporin resistance (n=17) was further examined. Final elastic net model identified 32 features associated with cluster membership compared to controls (n=213), including open excision 5–9 days prior (OR: 28.1, p< 0.001) and skin graft 2 days prior (OR: 45.2, p< 0.001).

**Conclusion:**

We demonstrate the use of EHRs to statistically detect related hospital-onset *P. aeruginosa* and suggest biologically plausible etiologies. Resistance-based models did not align with clusters confirmed by WGS. Future research will establish causal links and extend to other hospital-acquired pathogens.

**Disclosures:**

Kathryn DeSear, PharmD, Abbvie: Advisor/Consultant|Biomerieux: Advisor/Consultant|Cormedix: Speaking|GSK: Advisor/Consultant|Shionogi: Speaking

